# Fast Food Intake Increases the Incidence of Metabolic Syndrome in Children and Adolescents: Tehran Lipid and Glucose Study

**DOI:** 10.1371/journal.pone.0139641

**Published:** 2015-10-08

**Authors:** Golaleh Asghari, Emad Yuzbashian, Parvin Mirmiran, Behnaz Mahmoodi, Fereidoun Azizi

**Affiliations:** 1 Nutrition and Endocrine Research Center, Research Institute for Endocrine Science, Shahid Beheshti University of Medical Sciences, Tehran, Iran; 2 Department of Clinical Nutrition and Dietetics, Faculty of Nutrition Sciences and Food Technology, National Nutrition and Food Technology Research Institute, Shahid Beheshti University of Medical Sciences, Tehran, Iran; 3 Endocrine Research Center, Research Institute for Endocrine Sciences, Shahid Beheshti University of Medical Sciences, Tehran, Iran; University Heart Center Freiburg, GERMANY

## Abstract

The aim of the study was to evaluate the association between fast food consumption and incidence of metabolic syndrome (MetS) and its components among children and adolescents over a 3.6 year follow-up. Dietary data of 424 healthy subjects, aged 6–18 years, was collected using a valid and reliable food frequency questionnaire. Metabolic syndrome was defined according to the Cook et al criteria. Consumption of fast foods including hamburgers, sausages, bologna (beef), and fried potatoes was calculated and further categorized to quartiles. Multiple logistic regression models were used to estimate the incidence of MetS and its components in each quartile of fast food intake. The incidence of MetS was 11.3% after a 3.6 year follow up. In the fully adjusted model, compared to the lowest quartile of fast food intake, individuals in the highest had odds ratios of 2.96 (95% CI: 1.02–8.63; *P* for trend<0.001), 2.82 (95% CI: 1.01–7.87; *P* for trend = 0.037), and 2.58 (95% CI: 1.01–6.61; *P* for trend = 0.009) for incidence of MetS, hypertriglyceridemia, and abdominal obesity, respectively. No significant association was found between fast food intakes and other components of MetS. Fast food consumption is associated with the incidence of MetS, abdominal obesity, and hypertriglyceridemia in Tehranian children and adolescents.

## Introduction

Metabolic syndrome (MetS) is a collection of metabolic abnormalities, including abdominal obesity, hypertriglyceridemia, low high-density lipoprotein-cholesterol (HDL-C) concentrations, hypertension, and hyperglycaemia [1], which has strong association with the development of type 2 diabetes and cardiovascular morbidity and mortality in adults [2, 3]. Prevalence and incidence of MetS are increasing rapidly in children and adolescents and become a major public health challenge worldwide [4]. The etiology of the MetS is complex and determined by the interplay of both genetic and environmental factors [5].

One potentially important dietary factor is fast food consumption, which can be defined as convenience food bought in self-service or carry-out food facilities [6]. Increase in fast food consumption among children drastically increases the total energy intake [7]. Fast food has several inherent characteristics such as excessive portion size, with single large meals often approaching or exceeding individual daily energy requirements; palatability, emphasizing primordial taste preferences for added sugar, salt, and fat; high energy density and last, but not least, high glycaemic load [8].

To our knowledge, there were no studies available, evaluating the association of fast-food consumption with metabolic abnormalities in longitudinal design and data available from cross-sectional studies are limited [9–11]. Recent investigations have indicated that, higher ultra-processed food intakes are positively associated with prevalent MetS among Brazilian adolescents [11]. In addition, increased fast-food consumption was associated with higher insulin resistance [9, 10].

Therefore, we aimed to investigate the association between fast food intakes and incidence of MetS and its component after a 3.6 year follow up among children and adolescents.

## Materials and Methods

### Subjects

This study was conducted within the framework of the Tehran Lipid and Glucose Study (TLGS), a prospective community-based study being conducted among a representative urban population of Tehran with the aim of ascertaining the prevalence of non-communicable disease risk factors and developing healthier lifestyles [12]. In the TLGS, 15005 subjects, aged ≥ 3 y, were selected by random multistage cluster sampling. The baseline survey was a cross-sectional study conducted from 1999 to 2001, and surveys 2 (2002–2005), 3 (2006–2008), and 4 (2009–2011) were prospective follow-up surveys.

In the third survey of the TLGS (2006–2008), from among 12523 examined participants, 3462 were randomly selected for dietary assessment; of these 621 individuals were aged 6–18 y. For the current study, subjects who did not have complete data on physical activity, anthropometric, and biochemical variables (n = 29) and those who over- or underreported (n = 6) were excluded. Depending on the outcome variable under analysis, subjects who had MetS (n = 69), hypertension (n = 53), high triglycerides (TGs, n = 168), low HDL-C (n = 242), high fasting plasma glucose (FPG, n = 12), or abdominal obesity (n = 145) at baseline for individual analysis of MetS and its components incidence were also excluded. Some individuals fell into more than one exclusion category. After a mean 3.6 year follow-up, the final sample size varied by outcomes as follows: MetS (n = 424), hypertension (n = 439), high TGs (n = 347), low HDL-C (n = 290), high FPG (n = 476), and abdominal obesity (n = 327).

The design of this study was approved by the institutional ethics committee of the Research Institute for Endocrine Sciences (RIES), Shahid Beheshti University of Medical Sciences, and written informed consent was obtained from participants' parents.

### Measurements

Weight was measured, while participants were minimally clothed without shoes, using digital scales (Seca 707, Seca Corp., Hanover, MD; range 0.1–150 kg) and recorded to the nearest 100 g. Height was measured in a standing position without shoes, using a stadiometer, with shoulders in normal alignment. Body mass index (BMI) was calculated as weight (kg) divided by square of height (m^2^). Waist circumference (WC) was measured at the umbilicus, using a measuring tape without pressure to body surfaces and was recorded to the nearest 0.5 cm. To measure blood pressure, the participants remained seated for 15 minutes, when a qualified physician, using a standard mercury sphygmomanometer with the cuff placed on the right arm, measured blood pressure twice and mean values were documented.

Blood samples, at baseline and follow up, were drawn between 7:00 and 9:00 AM from all study participants, after 12–14 h overnight fasting; all blood analyses were done at the TLGS research laboratory on the day of blood collection. Fasting plasma glucose was measured by the enzymatic colorimetric method using glucose oxidase. Serum HDL-C was measured after precipitation of the apolipoprotein B-containing lipoproteins with phosphotungstic acid and serum TGs were assayed using an enzymatic colorimetric method with glycerol phosphate oxidase. These analyses were performed using commercial kits (Pars Azmoon Inc., Tehran, Iran) and a Selectra 2 auto analyzer (Vital Scientific, Spankeren, The Netherlands). Inter- and intra-assay coefficients of variations at baseline were both 2.2% for FPG, 2.0 and 0.5% for HDL-C and 1.6 and 0.6% for TGs, respectively.

### Dietary and physical activity assessment

A valid and reliable 168-item semi-quantitative food frequency questionnaire (FFQ) [13–15] was used by trained dietitians during face-to-face interviews to evaluate usual dietary intakes. Participants were asked to report their consumption frequency during the previous year on a daily, weekly, or monthly basis; data were then converted to the daily intakes. Mothers were asked about the type and quantity of meals and snacks when children were unable to recall. Portion sizes of consumed foods, reported in household measures, were specified according to the US Department of Agriculture (USDA) standard portion sizes and were then converted to grams. When using USDA portion sizes was impossible, household measures (e.g. beans, 1 tablespoon; chicken meat, 1 leg or wing; rice, 1 large or small plate) were used alternatively. In this analysis, the most commonly consumed fast foods in Iran, including sausage, bologna (beef), hamburger, pizza, and French fries were considered as fast foods. Total consumption of fast food for each subject was calculated by summing up the weekly consumption of these foods.

The Modifiable Activity Questionnaire (MAQ) was used to assess physical activity patterns among children and adolescents. High reliability (98%) and moderate validity (47%) were ascertained for the MAQ translated into Persian [16]. Individuals were asked to report the physical activities which they had participated in during the past 12 months, and to specify the frequency and duration for each activity identified. Each activity was weighted by its relative intensity, referred to as metabolic equivalent (MET). One MET represents the energy expenditure for an individual at rest (1 MET = 3.5 mL.kg−1.min−1 of O2 consumption). For all activity levels, obtained MET was multiplied by the time spent at each level. MET-time from each level was added to total 24 hour MET- time, representing the average daily level of physical activity. In the current study, vigorous physical activity level was categorized as MET ≥ 6.

### Definitions

Over- and under-reporting subjects were considered to be those in the top and bottom 1% of the reported energy intake to the estimated energy requirement ratio.

For children and adolescents, MetS was defined according to the criteria, proposed by Cook et al as having ≥3 of the following [17]: Fasting TGs ≥110 mg/dl; HDL-C<40 mg/dl; WC≥90^th^ percentile for age and sex, according to national reference curves [18]; systolic blood pressure and diastolic blood pressure ≥90^th^ percentile for sex, age and height, from the National Heart, Lung, and Blood Institute’s recommended cut-off points [19], and FPG≥100 mg/dl, according to the recommendations of American Diabetes Association [20]. For subjects who were >18 years old after follow-up, MetS was defined according to the joint interim statement [21] as the presence of any 3 of the following 5 risk factors: Abdominal obesity as WC ≥91cm for women and ≥89 cm for men according to Iranians cut-off point [22]; FPG ≥100 mg/dl or drug treatment; fasting TGs ≥150 mg/dl or drug treatment; fasting HDL-C <50 mg/dl for women and <40 mg/dl for men or drug treatment and hypertension as systolic blood pressure ≥130 mm Hg, diastolic blood pressure ≥85 mm Hg, or antihypertensive drug treatment.

### Statistical analysis

Statistical analysis was performed using the Statistical Package for Social Sciences (version 15.0; SPSS Inc, Chicago IL). Characteristics of subjects at baseline were expressed as mean±SD or median (interquartile 25–75) for continuous variables and percentages for categorical variables. To test for a trend across quartiles of fast food intakes, linear regression for continuous variables and Pearson Chi-square test for dichotomous variables were used. Nutrient and food group intakes were reported according to quartiles of fast food after energy intake adjustment and tested for a trend, using linear regression by assigning the median value for each quartile of intake treated as a continuous variable. The cumulative incidence of MetS was 11.3% (girls: 9.1%, boys: 14.4%), low HDL-C was 9.2% (girls: 5.8%, boys: 14.2%), abdominal obesity was 19% (girls: 18.1%, boys: 19.1%), high TGs was 14.1% (girls: 11.3%, boys: 17.6%), hypertension was 8.4% (girls: 7.8%, boys: 9.8%), and high FPG was 11.6 (girls: 9.4%, boys: 14.4%).

Subjects were categorized according to quartiles of fast food consumption as follows: <244, 244–384, 385–533, and ≥534g/week for quartiles one through four, respectively. Multiple logistic regression models were used to examine the association between the incidence of MetS and its components with fast food consumption and the odds ratios (OR) and 95% CIs were calculated. Age, sex, total energy intake, physical activity, dietary fiber, family history of diabetes, food groups (meat, poultry, fish, grains, and legumes), and BMI were included as confounders. P for trend was calculated from the logistic regression model using the median values for quartiles of fast food intakes as a continuous variable.

## Results

The mean±SD age of participants (57% girls) was 13.6±3.7 years. Mean energy intake from fast foods was 175 kcal/d out of 2220 total energy (~8%). There was an over 4-fold difference in fast food intake between the highest and lowest quartiles of subjects (median 776 g/wk in the highest quartile vs. 180 g/wk in the lowest). Mean weekly intakes of hamburger, sausage, bologna (beef), pizza, and French fries were 58.4, 137, 54.0, 116, and 121 g/wk, respectively. There was no significant difference in baseline anthropometric measurements and biochemical assessments between subjects followed up and those who were lost to follow up (data not shown).

Baseline anthropometric and metabolic characteristics of participants across quartiles of fast food intake are shown in Table 1. Adolescents in higher quartiles of fast food intakes were more likely to be boys and had increased WC and FPG. Subjects in the highest quartile of fast food intake also had lower intakes of carbohydrate, whole grain, and fish, but higher intakes of energy, cholesterol, refined grain, meat, poultry, and solid fat (Table 2).

**Table 1:** Baseline characteristics of participants according to quartiles of fast food consumption.

	Fast food intakes				*P*
	Q1 (n = 106)	Q2 (n = 106)	Q3 (n = 106)	Q4 (n = 106)	
Median (g/week)	180.5	304.7	448.1	776.6	
Age (years)	14.0±3.7	13.3±3.9	12.7±3.5	14.1±3.6	0.762
Girls (%)	64	69	54	52	0.048
Family history of diabetes (%)	5.7	3.8	3.8	7.5	0.756
Waist circumstance (cm)	67.5±10.9	68.7±10.4	69.1±10.5	69.5±10.0	0.043
Body mass index (kg/m^2^)	19.8±4.0	19.8±3.7	20.2±4.0	20.7±3.8	0.097
Systolic blood pressure (mmHg)	97.4±12.1	96.6±11.0	97.8±12.1	100.0±11.6	0.202
Diastolic blood pressure (mmHg)	63.3±9.9	65.7±8.2	63.3±9.6	66.2±10.4	0.140
Fasting plasma glucose (mg/dl)	83.7±6.2	85.1±5.6	85.3±5.9	85.7±6.6	0.019
High density lipoprotein (mg/dl)	47.1±12.0	45.5±11.5	45.8±9.6	45.0±8.9	0.955
Triglycerides (mg/dl)	75 (59–104)	83 (67–100)	77 (62–99)	84 (65–117)	0.162
Vigorous physical activity (%)	18	18	17	17	0.712

Data are represented as mean±SD or median (IQ 25–75) for continuous variables and percent for categorically distributed variables

*P* is based on linear regression for continuous and chi-square test for categorical variables

**Table 2:** Dietary intakes according to quartiles of fast food intake among children and adolescents at baseline: Tehran Lipid and Glucose Study.

	Fast Food intakes				*P* for trend
	Q1 (n = 106)	Q2 (n = 106)	Q3 (n = 106)	Q4 (n = 106)	
Median (g/week)	180.5	304.7	448.1	776.6	
Energy (kcal)	1926±729	2244±786	2727±1484	3326±1459	<0.001
Protein (% energy)	12.3±2.1	12.8±2.5	13.4±1.9	13.8±2.1	0.054
Carbohydrate (% energy)	58.0±8.4	57.1±7.2	57.0±5.8	55.9±7.2	<0.001
Fat (% energy)	32.1±7.9	32.5±6.7	32.0±5.6	32.6±7.4	0.102
Saturated fat (% energy)	11.2±3.4	10.8±2.7	11.4±3.1	11.0±2.9	0.405
Monounsaturated fatty acid (% energy)	11.1±3.2	11.4±3.2	10.2±2.1	11.2±2.8	0.820
Polyunsaturated fatty acid (% energy)	6.6±2.6	6.8±2.2	6.3±1.9	6.6±2.0	0.698
Dietary fiber (g/1000 kcal)	14.0±5.2	13.9±4.7	15.2±6.9	14.8±6.1	0.137
Cholesterol (mg/d)*	189±11	225±11	260±11	298±11	<0.001
Fruit (g/d)*	396±31	420±31	413±30	413±32	0.319
Vegetable (g/d)*	226±14	239±14	258±14	260±15	0.222
Whole grain (g/d)*	105±15	85±14	82±14	69±15	0.012
Refined grain (g/d)*	369±20	395±20	399±19	407±20	0.042
Legumes (g/d)*	20.6±2.3	18.4±2.19	15.5±2.2	14.2±2.3	0.054
Meat (g/d)*	24.4±3.4	32.3±3.2	31.1±3.2	43.1±3.4	0.001
Poultry (g/d)*	12.1±3.3	18.5±3.3	23.0±3.2	49.9±3.4	<0.001
Nuts (g/d)*	10.3±1.3	10.1±1.3	7.1±1.3	11.8±1.4	0.901
Fish (g/d)*	26.2±7.3	15.2±7.1	11.6±7.1	3.54±7.48	<0.001
Dairy (g/d)*	539±36	497±35	615±43	476±36	0.823
Liquid oil (g/d)*	6.4±0.78	7.2±0.76	7.1±0.76	6.5±0.80	0.503
Solid fat (g/d)*	23.8±2.3	24.4±2.4	25.2±2.3	26.3±2.4	<0.001

Data are represented as mean±SD or mean±SE for energy adjusted variables

*P* for trend is based on linear regression model using the median values for quartile of fast food intakes as a continuous variable

*Energy adjusted

After adjustment of age, sex, total energy intake, physical activity, dietary fiber, family history of diabetes, food groups (meat, poultry, fish, grains, and legumes), and BMI, the ORs of incident MetS across increasing quartiles of fast food were 1.00, 1.35, 1.56, and 2.96 (*P* for trend<0.001). The adjusted ORs of incident abdominal obesity were 1.00, 0.70, 1.02, and 2.58 from the lowest to the highest quartiles of fast food (*P* for trend = 0.009). Also, the adjusted ORs for incidence of high TGs across increasing quartiles of fast food intake were 1.00, 1.17, 1.54, and 2.82 (*P* for trend = 0.037; Table 3).

**Table 3:** Multivariable-adjusted odds ratio (95% CIs) for incident MetS and its components according to quartiles of fast food intake, among children and adolescents during a 3.6-year follow-up.

	Fast food intakes				*P* for trend
	Q1	Q2	Q3	Q4	
Median (g/week)	180.5	304.7	448.1	776.6	
**Metabolic syndrome (n)**	106	106	106	106	
Model 1	1.00	1.29 (0.43–3.85)	1.62 (0.57–4.72)	2.58 (0.89–7.45)	0.001
Model 2	1.00	1.35 (0.45–4.05)	1.56 (0.53–4.59)	2.96 (1.02–8.66)	<0.001
**Low HDL-C (n)**	72	73	73	72	
Model 1	1.00	1.76 (0.48–6.43)	1.81 (0.47–6.92)	1.39 (0.38–5.42)	0.344
Model 2	1.00	2.14 (0.56–8.24)	2.02 (0.51–7.94)	1.76 (0.43–7.20)	0.443
**Abdominal obesity (n)**	82	82	81	82	
Model 1	1.00	0.87 (0.37–2.05)	1.09 (0.48–2.47)	1.89 (0.83–3.32)	0.005
Model 2	1.00	0.70 (0.27–1.80)	1.02 (0.41–2.58)	2.58 (1.01–6.61)	0.009
**Hypertension (n)**	110	110	109	110	
Model 1	1.00	1.05 (0.35–3.12)	1.14 (0.39–3.32)	1.55 (0.51–4.66)	0.323
Model 2	1.00	1.16 (0.38–3.51)	1.16 (0.39–3.42)	1.62 (0.53–4.96)	0.318
**High fasting plasma glucose (n)**	119	119	119	119	
Model 1	1.00	1.39 (0.61–3.176)	1.10 (0.41–2.50)	1.13 (0.42–3.01)	0.786
Model 2	1.00	1.41 (0.61–3.22)	1.02 (0.41–2.49)	1.15 (0.43–3.05)	0.645
**High triglycerides (n)**	86	87	87	87	
Model 1	1.00	1.15 (0.42–3.16)	1.61 (0.60–4.31)	2.41 (0.88–6.57)	0.059
Model 2	1.00	1.17 (0.42–3.25)	1.54 (0.57–4.18)	2.82 (1.01–7.85)	0.037

Model 1: Adjusted for age, sex, total energy intake, physical activity, dietary fiber, family history of diabetes, and food groups (meat, poultry, fish, grains, and legumes)

Model 2: Additionally adjusted for body mass index

*P* for trend is based on logistic regression model using the median values for quartile of fast food intakes as a continuous variable

Fully adjusted ORs for incident MetS were 2.59, 2.64, and 2.82 comparing subjects in the highest quartile to those in the lowest quartile of sausage (*P* for trend = 0.004), French fries (*P* for trend = 0.009), and pizza (*P* for trend = 0.018) intakes, respectively (Table 4).

**Table 4:** Multivariable-adjusted odds ratio (95% CIs) for incident metabolic syndrome according to quartiles of fast food items, among children and adolescents during 3.6-year follow-up.

	Fast food intakes				*P* for trend
	Q1 (n = 106)	Q2 (n = 106)	Q3 (n = 106)	Q4 (n = 106)	
**Hamburger (g/week)**	12.95	26.00	39.00	103.97	
Model 1	1.00	1.20 (0.44–3.24)	1.58 (0.59–4.18)	2.17 (0.91–5.22)	0.074
Model 2	1.00	1.23 (0.45–3.35)	1.60 (0.60–4.24)	2.21 (0.92–5.32)	0.067
**Sausages (g/week)**	19.83	84.98	127.50	223.48	
Model 1	1.00	0.89 (0.32–2.51)	0.92 (0.28–3.02)	2.64 (1.09–6.37)	0.004
Model 2	1.00	0.83(0.29–2.36)	0.91 (0.28–3.05)	2.59 (1.07–6.28)	0.004
**Bologna (beef) (g/week)**	2.00	17.01	43.65	113.75	
Model 1	1.00	1.62 (0.15–6.92)	1.67 (0.84–3.32)	2.27 (0.81–5.11)	0.162
Model 2	1.00	1.69 (0.15–6.99)	1.72 (0.86–3.42)	2.34 (0.87–5.23)	0.143
**French fries (g/week)**	16.95	50.86	72.67	281.01	
Model 1	1.00	1.03 (0.32–3.29)	0.99 (0.36–2.72)	2.43 (0.95–6.19)	0.006
Model 2	1.00	1.05 (0.32–3.40)	1.03 (0.37–2.84)	2.64 (1.02–6.83)	0.009
**Pizza (g/week)**	24.92	53.40	106.80	213.00	
Model 1	1.00	1.35 (0.44–4.11)	1.98 (0.71–5.52)	2.61 (0.94–7.21)	0.023
Model 2	1.00	1.38 (0.45–4.26)	2.08 (0.75–5.83)	2.82 (1.01–7.88)	0.018

Model 1: Adjusted for age, sex, total energy intake, physical activity, dietary fiber, family history of diabetes, and food groups (meat, poultry, fish, grains, and legumes)

Model 2: Additionally adjusted for body mass index

*P* for trend is based on logistic regression model using the median values for quartile of fast food intakes as a continuous variable

## Discussion

In the current study, children and adolescents showed undesirable effects of fast food intakes on incidence of MetS. In addition, positive associations were found between fast food consumption and increased risk of abdominal obesity and hypertriglyceridemia. Sausage, French fries, and pizza consumption were associated with higher risk of incident MetS.

Our findings are in agreement with those of cross-sectional studies of fast food and MetS [9, 11]. Tavares et al [11] reported that high consumption of ultra-processed foods was associated with higher prevalence of MetS in adolescents. Additionally, recent evidence indicated that higher intake fast food and greater access to the fast foods may lead to higher insulin levels and risk of insulin resistance [9, 10].

Data from previous studies revealed the undesirable effects of fast food consumption on overweight, obesity, and cardio-metabolic risk factors [23–25]. Among Isfahanian children, overweight and obese adolescents consumed fast foods 2.7 times/week, compared to 1.2 times/week in their normal-weight counterparts [23]. In addition, among 4–19 year-old American children and adolescents participating in a nationally representative study, fast-food consumption was found to increase the risk of obesity [25]. A significant association was also reported between dyslipidemia and the intake frequencies of hydrogenated fat, fast foods, cheese puffs, and potato chips among 11–18 year-old students from urban and rural areas of two provinces in Iran [24].

Fast food consumption is a component of poor diets. As demonstrated by studies, increasing fast food intake is accompanied by higher intakes of carbohydrate, cholesterol, refined grain, meat, poultry and lower consumption of whole grains; fast-food consumption among students is reported to be associated with higher intakes of French fries and soft drinks and lower intakes of fruit, vegetable, and milk [26]. Poor diet quality of fast food dietary patterns [27], and dietary factors such as grains, red meat, trans- and saturated fats as well as high energy density have been reported as influential factors in the development of insulin resistance and MetS [28, 29].

Industries add high amounts sodium, solid fats, and sugars as flavor enhancement strategies of fast foods formulation. Fast foods have high glycemic index and energy density, because they contain excessive amounts of dietary fats, salt, trans- and saturated fatty acids, and low levels of dietary fiber. In Iranian fast foods, trans fatty acids constitute 23.6% to 30.6% of total fatty acids [30], compared to the recommended amounts of <10% of calories from saturated fatty acids and low levels of trans fatty acids [31]. Children who eat fast foods, compared to those who do not, consume more total energy (187 kcal), energy/g of food (0.29 kcal/g), total fat (9 g), total carbohydrate (24 g), added sugars (26 g), sugar-sweetened beverages (228 g), and less fiber (−1.1 g), milk (−65 g), and fruits and non-starchy vegetables (−45 g) [25]. Another important factor is the portion sizes of burgers, fried potatoes, pizzas, and soft drinks at fast-food outlets, all of which have increased 2–5-fold over the last 50 years [32]. A study that investigated fatty acid contents of foods (French fries and fried chicken) in McDonald and KFC outlets in 35 countries between 2005–2006, found that in 160 g of chicken meat and 171 g of French fries, the total fat content varies from 41 to 65 g at McDonald’s and from 42 to 74 g at KFC; their trans fat content varies from 0.3 to 10.2 and 0.3 to 24 g, respectively [33].

Expression and secretion of inflammatory cytokines, such as IL-6 and CRP are increased by higher intakes of saturated and trans fatty acids [34, 35]. Inflammation and disturbed metabolic homeostasis of the body may contribute to a family of disorders including insulin resistance, glucose intolerance, hyperlipidemia and hypertension, collectively known as metabolic syndrome [36].

Our study has several noteworthy limitations. The puberty stage was not considered in our analysis although it is evident that MetS components may undergo variations during puberty and alterations occur with maturity; notwithstanding the compensatory increase in insulin secretion and decrement of insulin sensitivity during puberty returns to normal at the end of puberty. In addition, systemic inflammation markers were not measured. These measurements would elucidate the pathways through which fast foods cause metabolic abnormalities. Despite these limitations, to our knowledge, this is the first study to evaluate fast food intakes in relation to incidence of MetS over a 3.6 year follow-up in the Middle East and North Africa, among children and adolescents and the results may provide valuable findings for development of strategies to reduce the prevalence of risk factors of chronic disease in adulthood. The longitudinal design of the study helped to confirm the role of fast food intakes in the incidence of cardio-metabolic risk factors. Furthermore, the socio-demographic information of the population-based cohort subjects had been collected in detail, facilitated a thorough investigation of possible confounders.

With globalization, Western-style fast food intake in developed and recently developing areas worldwide [37, 38] is becoming fast more common because of easy access, familiarity and global advertising [39]. In addition, Western-style fast food intake in Asia, Australia, Middle East and North Africa (MENA) is fast becoming popular as it is in the USA [40–42]. Simultaneously, with increasing consumption of fast food and soft drinks worldwide, nutrition related non communicable diseases (NR-NCD) have truly become a global burden, affecting both the wealthy and poor countries. Research on cross-cultural factors contributing to fast rising incidence of NR-NCD may provide insight into critical global public health issues.

In conclusion, the results of this study indicate that higher intakes of fast food have undesirable effects on the incidence of MetS, hypertriglyceridemia, and abdominal obesity, a finding which requires prioritized health strategies aimed at prevention of cardio-metabolic risk factors in children and adolescents.
